# Motion and twisting of magnetic particles ingested by alveolar macrophages in the human lung: effect of smoking and disease

**DOI:** 10.1186/1477-044X-4-4

**Published:** 2006-05-15

**Authors:** Winfried Möller, Winfried Barth, Martin Kohlhäufl, Karl Häussinger, Wolfgang G Kreyling

**Affiliations:** 1 GSF National Research Center for Environment and Health, Clinical Research Group 'Inflammatory Lung Diseases' and Institute for Inhalation Biology, Gauting, Germany; 2 GSI National Research Center for Heavy Ions, Darmstadt, Germany; 3 Asklepios Hospital Munich-Gauting, Center for Respiratory Medicine, Gauting, Germany

## Abstract

**Background:**

Magnetic microparticles being ingested by alveolar macrophages can be used as a monitor for intracellular phagosome motions and cytoskeletal mechanical properties. These studies can be performed in the human lung after voluntary inhalation. The influence of cigarette smoking and lung diseases on cytoskeleton dependent functions was studied.

**Methods:**

Spherical 1.3 *μ*m diameter ferrimagnetic iron oxide particles were inhaled by 17 healthy volunteers (40 – 65 years), 15 patients with sarcoidosis (SAR), 12 patients with idiopathic pulmonary fibrosis (IPF), and 18 patients with chronic obstructive bronchitis (COB). The retained particles were magnetized and aligned in an external 100 mT magnetic field. All magnetized particles induce a weak magnetic field of the lung, which was detected by a sensitive SQUID (superconducting quantum interference device) sensor. Cytoskeletal reorganizations within macrophages and intracellular transport cause stochastic magnetic dipole rotations, which are reflected in a decay of the magnetic lung field, called relaxation. Directed phagosome motion was induced in a weak magnetic twisting field. The resistance of the cytoplasm to particle twisting was characterized by the viscosity and the stiffness (ratio between stress to strain) of the cytoskeleton.

**Results:**

One week after particle inhalation and later macrophage motility (relaxation) and cytoskeletal stiffness was not influenced by cigarette smoking, neither in healthy subjects, nor in the patients. Patients with IPF showed in tendency a faster relaxation (p = 0.06). Particle twisting revealed a non-Newtonian viscosity with a pure viscous and a viscoelastic compartment. The viscous shear was dominant, and only 27% of the shear recoiled and reflected viscoelastic properties. In patients with IPF, the stiffness was reduced by 60% (p < 0.02). An analysis of the shear rate and stress dependence of particle twisting allows correlating the rheological compartments to cytoskeletal subunits, in which microtubules mediate the pure viscous (non-recoverable) shear and microfilaments mediate the viscoelastic (recoverable) behavior. The missing correlation between relaxation and particle twisting shows that both stochastic and directed phagosome motion reflect different cytoskeletal mechanisms.

**Conclusion:**

Faster relaxation and a soft cytoskeleton in patients with IPF indicate alterations in cytoskeleton dependent functions of alveolar macrophages, which may cause dysfunction's in the alveolar defense, like a slower migration, a retarded phagocytosis, a disturbed phagosome lysosome fusion and an impaired clearance.

## Background

The lungs with about 140 m^2 ^surface area [1] represent the organ of the human body with the biggest contact area to the environment. Every breath transports many environmental particles into the lungs, which are partially removed and deposited. The different areas of the lung have different mechanisms of deposition and clearance [2]. Clearance from the airway is primarily due to mucociliary transport, where most particles are removed within 24 h from the lungs. The alveolar region is not covered by a mucus layer. Alveolar macrophages are resident in each of the alveoli and phagocytize foreign materials. Phagocytosis transfers the microparticles into phagosomes of the macrophage. After fusion with lysosomes ingested particles, bacteria or viruses are digested due to acidic pH within phagolysosomes and the release of reactive substances, such as oxygen radicals [3-5]. The cytoskeleton of AM is crucially involved in these defense reactions, including locomotion and cell migration, phagocytosis, intracellular transport, phagosome-lysosome fusion and signal transduction [6-8].

Magnetopneumography (MPG) uses ferrimagnetic iron oxide particles as a tracer to investigate the long-term clearance from the alveolar region of the human lungs [9-12]. These particles are non-toxic and non-cancinogenic [13], and can be inhaled and detected by sensitive magnetic field sensors. Because of the chemical stability, these particles can be used as a probe for intracellular phagosome motions and cytoskeletal integrity. Permanent cytoskeletal reorganizations and the intracellular transport cause a randomization of the magnetic dipole particles, which is reflected in a decay of the magnetic lung field and is called relaxation. Relaxation is an *in vivo *measure of macrophage motility, which is important in several defense reactions, like cell migration, phagocytosis, phagosome – lysosome fusion etc. Directed (external) phagosome motions are induced in a weak magnetic twisting field in order to investigate the mechanical properties of the cytoskeleton. Application of continuous and discrete twisting stress yields the rheological properties of the cytoskeleton, which reflects the integrity of the cytoskeleton and the role of the different cytoskeletal units in phagosome transport.

Previously, we have developed protocols to study cytoskeleton associated functions in macrophages, such as phagosome transport, mechanical cytoskeletal integrity and phagocytosis, using ferromagnetic microparticles, *in vitro *in cell cultures and *in vivo *after voluntary inhalation [14-17]. Magnetic twisting cytometry (MTC) has been used to investigate the role of the different cytoskeletal structures in macrophage function after application of selectively acting cytoskeletal drugs (Cytochalasin D, Nocodazole; [16]). Additionally it has been shown that MTC can monitor cytotoxicity of GaAs-particles *in vivo *in lung macrophages of animals [18,19] and of ultrafine particles in vitro [20,21].

The aim of this study was first, to investigate stochastic and directed phagosome motions in healthy subjects with respect to *in vivo *reactions of macrophages to a chronic cigarette smoke exposure, which was evident from *in vitro *studies [22-24]. The second objective was to verify whether reactions seen in smokers accumulate in patients with chronic obstructive bronchitis (COB), which is primarily the result of a long cigarette smoking history, and third, to correlate phagosome motions with interstitial lung diseases, like sarcoidosis (SAR) and idiopathic lung fibrosis (IPF), where the ethiology of the disease is not known. It was proposed that a chronic inflammation in the lungs of these patients might influence the phagosome transport and the cytoskeletal integrity.

## Methods

### Subjects and pulmonary function testing

A group of healthy subjects was studied (Table 1) with 40 to 65 years of age (12 men, five women), which was divided into never smokers (NS) and asymptomatic smokers (S). Further, 18 outpatients with stable chronic obstructive bronchitis (COB) (10 men and 8 women; 45 to 72 years of age), 12 outpatients with idiopathic pulmonary fibrosis (IPF) (five men and seven women; 27 to 74 years of age), and 15 outpatients with pulmonary sarcoidosis (SAR) (five men and ten women; 34 to 69 years of age) participated in this study. Chronic bronchitis was defined as cough and sputum production occurring on most days of the month for at least three months/year during the two years prior to the study [25]. Among COB patients, more than 50% were no longer active smokers and were therefore classified into a separate class of ex-smokers (XS). Anamnesis was carried out using a questionnaire based on ATS – recommendations [26]. The smoking history of the participants was quantified using the cumulative cigarette consumption expressed in pack-years (PY). None of the healthy subjects had a history of respiratory or cardiovascular disease or was receiving any long-term medication. Only smokers with normal lung function data were enrolled in the study. 6 patients with COB were receiving long-term *β*_2_-agonists and inhaled corticosteroids. 3 patients with COB received no medication at the time of the study. 4 patients with IPF and 2 patients with SAR were treated with oral steroids. The protocol was approved by the Ethical Committee of the Medical School of the Ludwig-Maximilian University (Munich, Germany), and informed consent from each subject was obtained.

**Table 1:** Age, cumulative cigarette smoke consumption, and lung function data of all subjects of the study.

	Healthy	SAR	IPF	COB
AGE (years)	54 +/- 7	48 +/- 14	49 +/- 15	60 +/- 8
NS/S/XS	9/8/0	10/2/3	5/4/3	1/3/14
Pack-years (PY)	49 +/- 18	18 +/- 13	15 +/- 6	40 +/- 25
FEV1 (%pred.)	105 +/- 15	93 +/- 15 (*)	82 +/- 23 (*)	72 +/- 28 (**)
FEV1/VC (%pred.)	91 +/- 10	94 +/- 6 (ns)	94 +/- 15 (ns)	69 +/- 19 (**)
R_AW _(kPaL^-1^s^-1^)	0.22 +/- 0.08	0.16 +/- 0.1 (ns)	0.22 +/- 0.14 (ns)	0.28 +/- 0.16 (ns)
RV % TLC	32 +/- 6	33 +/- 6 (ns)	37 +/- 12 (ns)	45 +/- 10 (**)
TGV (%pred.)	112 +/- 25	89 +/- 23 (*)	91 +/- 33 (ns)	119 +/- 34 (ns)
VC (%pred.)	118 +/- 13	102 +/- 17 (**)	86 +/- 19 (**)	103 +/- 25 (ns)
MEF_75 _(%pred.)	107 +/- 35	92 +/- 17 (ns)	90 +/- 45 (ns)	52 +/- 37 (**)
MEF_50 _(%pred.)	82 +/- 27	75 +/- 24 (ns)	71 +/- 40 (ns)	32 +/- 25 (**)
MEF_25 _(%pred.)	62 +/- 27	48 +/- 22 (ns)	51 +/- 27 (ns)	22 +/- 18 (**)
T_L,CO _(%pred.)	107 +/- 33	98 +/- 14 (ns)	82 +/- 13 (**)	92 +/- 21 (ns)

Relative values of lung function parameters are given as a percentage of predicted values (% pred., [27]). Values are presented as mean +/- SD. Healthy: healthy subjects, age 40 – 65 years, SAR: patients with sarcoidosis, IPF: patients with idiopathic pulmonary fibrosis, COB: patients with chronic obstructive bronchitis. FEV_1_: forced expiratory volume in one second, VC: vital capacity, R_aw_: airway resistance, RV: residual volume, TLC: total lung capacity, TGV: thoracic gas volume, MEF_25_, MEF_50_, MEF_75_: maximal expiratory flow at 25, 50 and 75% of VC, respectively, T_L,CO_: transfer factor of the lung for carbon monoxide; ns: not significant; *: p < 0.05; **: p < 0.01.

Body plethysmography and spirometry were performed using a Jäger Masterlab (Erich Jäger, Würzburg, Germany). Relative values of conventional lung parameters were calculated by normalizing to the reference values proposed by the European Community for Steel and Coal [27]. The transfer factor of the lung for carbon monoxide (T_L,CO_) was calculated as proposed by Cotes [28]. A lung function test and the MPG measurement of the natural ferromagnetic contamination of the lungs of every subject were obtained before inhalation. MPG measurements were performed 30 min., two days, one week, one month, five months and nine months after particle inhalation.

### Preparation of ferrimagnetic tracer-particles, inhalation and detection

The preparation, inhalation and detection of the magnetic particles is described in detail elsewhere [11]. In brief spherical monodisperse ferrimagnetic iron-oxide particles (Fe_3_O_4_, 2.9 *μ*m aerodynamic, 1.35 *μ*m geometric diameter, *σ*_g _< 1.1) were produced by a Spinning Top Aerosol Generator (STAG) [29], concentrated by using a virtual impactor, and directly inhaled by the participants under standard conditions (250 cm^3^/s flow rate, 1 L tidal volume). After inhalation, the subject lies on a bed with the lungs directly under the magnetizing coils. Magnetization is performed by discharging a capacitor battery into a 40 cm diameter copper coil. After the magnetizing current has decayed, the magnetized particles produce a weak remanent magnetic lung field (LF) of about 100 pT, which was detected by moving the subject under a superconducting magnetic field sensor (SQUID, superconducting quantum interference device). Particle twisting was performed by a weak magnetizing current in the 40 cm diameter copper coil, which was controlled for discrete time durations.

For MPG measurements about 1 mg of iron oxide particles were deposited in the lung. This amount is low compared to occupational exposure at work places, such as welders [30,31]. In a previous study a bronchoalveolar lavage was performed in one subject 7 days after particle inhalation [32]. In this study only single particles could be found in the alveolar macrophages, confirming the rotational behavior of single particles in cells.

### Correction for non-rotational particles (phagocytosis)

During inhalation, the particles are deposited on the epithelial surface of the lungs. Over the following 24 hours, they are phagocytized by alveolar macrophages. Directly after inhalation, particle twisting in a weak magnetic field reveals an alignment asymmetry, which reflects a certain fraction of non-rotatable particles [17]. After the particles are pulse magnetized, they are aligned with the magnetizing field. Then they are twisted by a reverse magnetic field into the opposite direction. When again reversing the twisting field, the particles are rotated to the initial direction of pulse magnetization. Free particles stack on the epithelium, are unable to follow this rotation and induce an asymmetry in the particle alignment, which allows estimating the fraction of non-rotatable particles. Directly after inhalation (30 min.), about 50% of the particles are not rotatable. This fraction decreases in the following hours to 5 – 10%. We suggest that the non-rotatable particles are not phagocytized by macrophages and stick to the alveolar epithelium. *In vitro *studies with J774 macrophages have shown that all particles which are phagocytized by macrophages are rotatable in weak twisting fields. Microscopic investigations have shown that these particles are covered by surfactant, displacing them into the epithelial lineage [33]. This tight contact to the epithelium hinders the particle rotation for a complete alignment. This *in vivo *measurement of non-rotatable particles measures the phagocytosis process in the human lungs. All relaxation and particle twisting data are corrected by the fraction of non-rotatable particles.

### Macrophage motility (relaxation)

**Figure 1:**
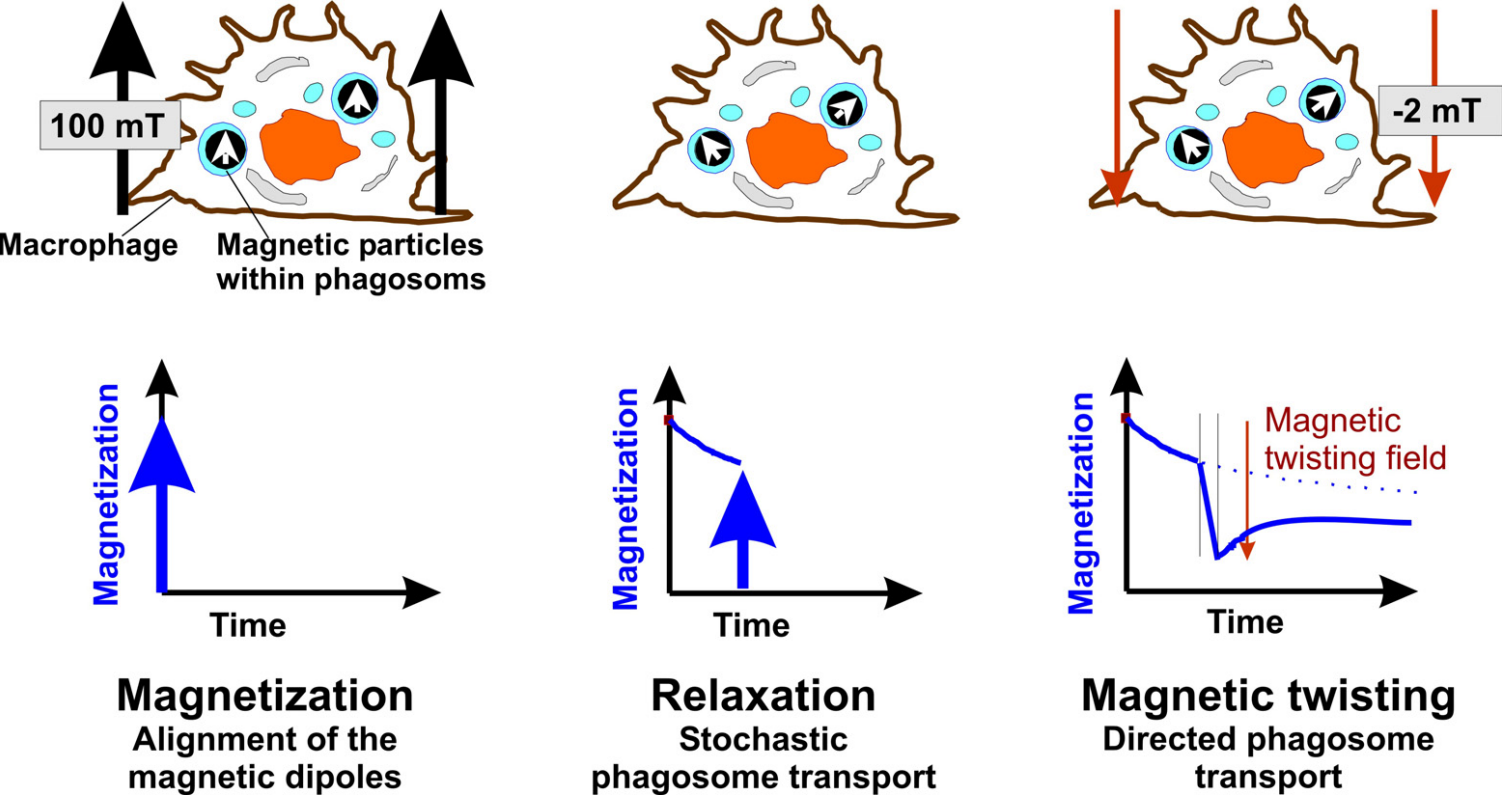
Schematic views of magnetic particles within macrophages during the three stages of the investigation showing the direction and strength of the magnetic field, the orientation of the dipoles formed by the magnetized particles, and the decay of the measured magnetic lung field (LF) over time.

**Figure 2:**
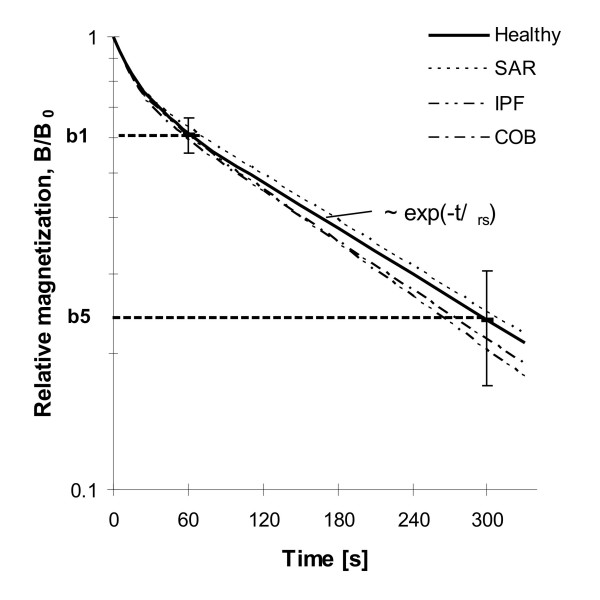
Measurement of macrophage motility as decay of the magnetic lung field (relaxation) after particle alignment by pulse magnetization. b1 = B(1 min.)/B_0 _and b5 = B(5 min.)/B_0 _denote the relative decay of the initial phase (after 1 min.) and of the slow phase (after five min.). The curves represent the mean relaxation behavior in healthy subjects, of patients with sarcoidosis (SAR), with interstitial lung fibrosis (IPF), and with chronic obstructive bronchitis (COB).

The motion of vesicles and phagosomes happens permanently within living cells and is part of the intracellular transport system. Figure 1 summarizes the phagosome motions, which can be followed in MPG. First, the particles are magnetized and aligned in a strong magnetic field pulse. Stochastic phagosome motions are caused by the intracellular transport mechanism and imply a decay of the remanent magnetic field of the lung (LF). This decay of the LF was called relaxation and was analyzed by the relative LF after 1 min., b1 = B(1 min.)/B_0_, which characterizes the initial fast phase, and the relative LF after 5 min., b5 = B(5 min.)/B_0_, being characteristic for the following slow phase (Figure 2). These two relaxation parameters are independent of any model. Modeling relaxation as a rotational Brownian motion process in a Newtonian fluid reveals an exponential decay (see Appendix). Relaxation in living cells shows deviations from this model with an initially faster decay, which can result from viscoelasticity [34,35]. For simplicity exponential functions were fitted to the initial and to the slow relaxation phase with the appropriate time constants.

### Macrophage motility (randomization energy)

**Figure 3:**
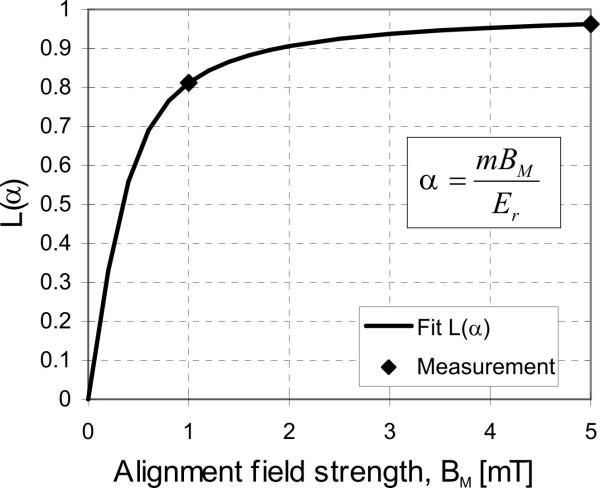
Measurement of cellular energy E_r _as competition between ordering of the dipoles in the external magnetizing field B_M _and randomization of the dipoles due to cytoskeletal motility.



We postulate a cellular energy E_r_, which is the driving force of intracellular transport mechanisms and of relaxation. During particle twisting this energy acts against the magnetic aligning force and prevents a complete orientation of the particles, as will be achieved in primary (strong field) magnetization. The balance between magnetic twisting force and cellular randomization force determines an equilibrium alignment of the particles in the cells (Figure 3). This equilibrium alignment has an analogy in paramagnetism and can be described by the so-called Langevin-function L(*α*) [36]:

where m is the magnetic dipole moment and B_M _is the external weak twisting field. This allows a direct measure of E_r_. The experiments were performed in a way that first, all particles were magnetized and aligned in a strong magnetic field, yielding the maximum lung field (B_max_). Relaxation was allowed over a period of 10 min., during which the lung field decayed below 50% of the initial value. Then a weak field of 1 mT strength was applied over 3 min. in order to realign the particles. The equilibrium lung field (B_eq_) is a measure of the achieved dipole alignment. Cellular energy E_r _was then estimated from *B*_*eq *_(*B*_*M*_) = *B*_max_·*L*(*α*). This method of estimating cell motility is independent of the viscoelastic environment.

### Cytoskeletal integrity: mechanical properties of the cytoskeleton of macrophages

Application of a weak magnetic field mediates an external particle twisting (Figure 1) and allows the investigation of mechanical properties of the cytoskeleton, like cytoplasmic rheology and mechanical integrity. Experiments for both discrete and continuous particle twisting were performed. Discrete particle twisting implies the force application for a short time period of 10 sec. This causes an angular shear of the particles and a measure of apparent viscosity. If the surrounding medium has elastic properties, we get elastic recoil which yields information about substructures of the cytoskeleton. During continuous particle rotation, the twisting field B_M _is applied for 3 min. until the dipole orientation reaches equilibrium.

Brownian rotational particle motion has an influence on particle twisting in a weak magnetic field, and, as was shown above, it prevents complete particle alignment. The influence is significant when the twisting force is small, as we have in small twisting fields, or when the magnetic dipole aligns with the twisting field, which is the case at continuous particle twisting during the equilibrium phase. For the estimation of viscous and elastic parameters, the influence of Brownian motion was neglected.

### Continuous particle twisting

**Figure 4:**
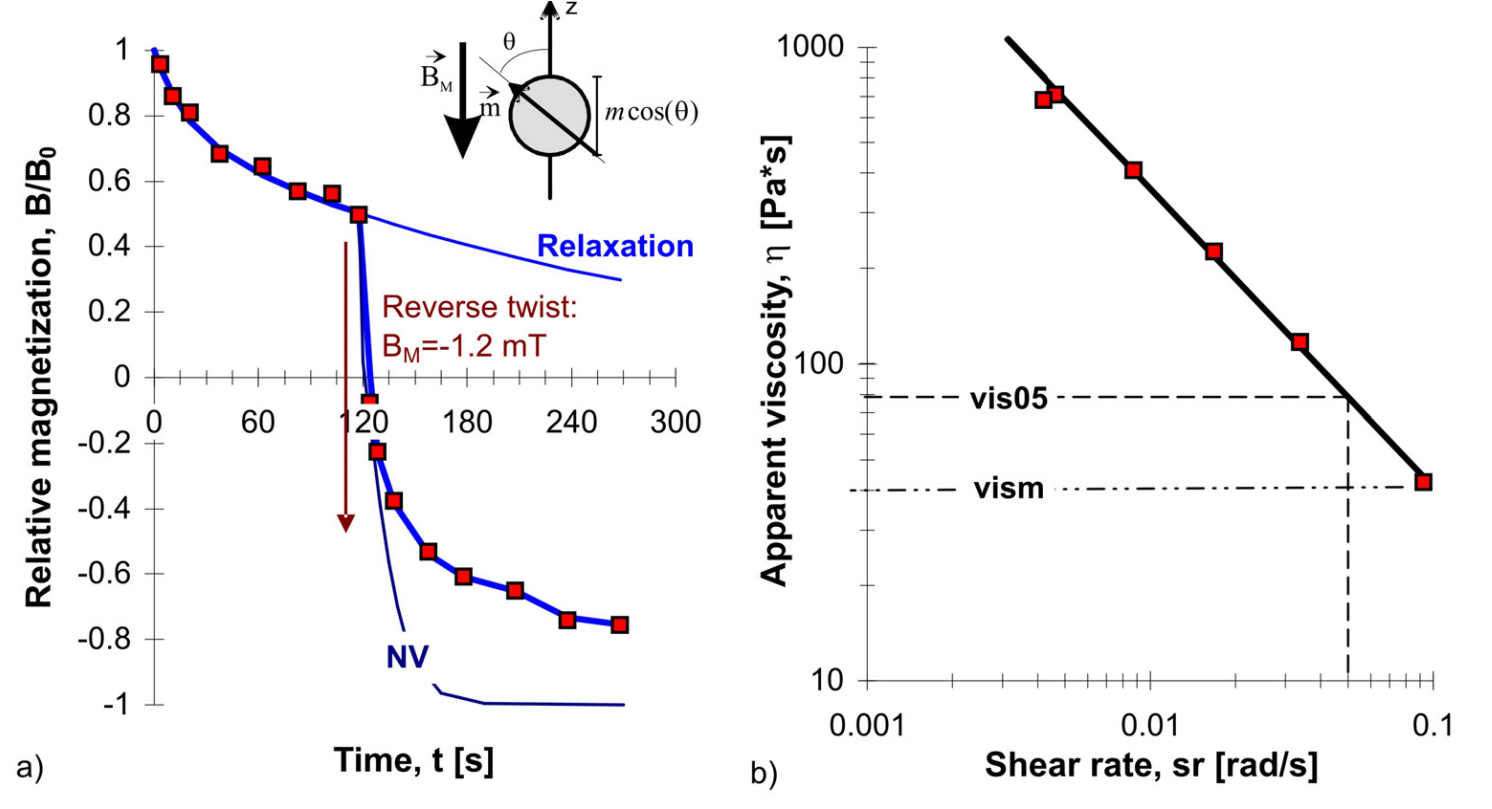
a) Continuous twist of the magnetic microparticles after pulse field alignment and 2 min. of relaxation. b) Analysis of the corresponding shear rate dependence of apparent viscosity during continuous particle twist. ηm denotes the apparent viscosity at the initial phase of particle twist, η5 denotes the apparent viscosity at a shear rate of 0.05 rad/s. The inset shows the a magnetic dipole, the component of detection along the z-axis and the rotation in the reverse twisting field.



The experimental procedure of continuous particle twisting is shown in Figure 4. After strong field magnetization, 2 min. relaxation was allowed in order to rotate the dipoles a certain angle away from the initial orientation. Then a reverse twisting field was applied for discrete time periods (5 s, 10 s, 20 s, 30 s etc.), between these periods, the LF was detected by moving the subject under the SQUID-sensor and back to the magnetizing coil, which requires about 3 – 4 sec. The small amount of elastic recoil during this short time period was neglected. This procedure was continued until a total twisting duration of 3 min. was achieved. Because the LF is a measure of cos*θ*(t) (see Appendix), we can get an estimation of the mean orientation angle *θ*(t) of the particles. Particles suspended in a Newtonian viscosity *η *rotate in an external magnetic twisting field according to Newton's law:

where d*θ*/dt is the shear rate, *σ *is the applied shear stress. The viscosity *η *is the constant of proportionality. The complete course of particle twisting under certain boundary conditions is described in detail in the Appendix.

The curve NV in Figure 4a shows particle twisting in a Newtonian viscosity (*η*_m_), and the time constant was adapted to the initial measurements. Increasing duration of stress application reveals a retarded particle rotation in the cells, which implies stiffening and non-Newtonian behavior of the cytoskeleton. The non-Newtonian apparent viscosity is characterized by the shear rate dependence according to Eq. A6 and is shown in Figure 4b. Apparent viscosity increases with decreasing shear rate sr according to a power law: *η *~ sr^-*α*^, where the power *α *is near unity. This behavior is called pseudoplastic [37] and is characteristic for particle twisting in alveolar macrophages and in J774 macrophages [38]. Simulations have shown that this behavior originates in part by elastic properties [15]. The previous analyses show that the first measure of apparent viscosity after 5 sec. stress duration (*η*_m_) is a reliable estimation of the viscosity of a more complex viscoelastic system.

### Discrete particle twisting (viscoelastic recoil)

Continuous particle twisting experiments demonstrate non-Newtonian viscous properties, but do not allow a direct measure of cytoskeletal elasticity. A visualization of cytoskeletal elasticity was achieved by applying the twisting field for a short time period of 10 sec. According to Hoke's law the applied shear stress is proportional to the strain (rotation angle *θ*), and the constant of proportionality is the rigidity *ν*.

*σ *= *ν*·*θ *    (3)

**Figure 5:**
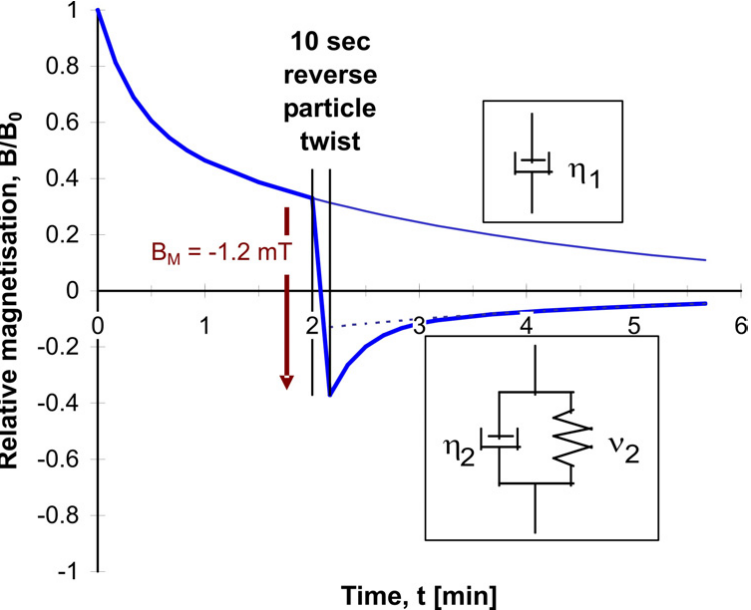
Discrete reverse particle twist after pulse field alignment and 2 min. of relaxation. The pure viscous element (viscosity *η*_1_) describes the non-recoverable strain and the viscoelastic element (viscosity *η*_2_, elastic modulus *ν*_2_) describes the elastic recoil behavior of the magnetic microparticles within macrophages.

Energy being stored during this shear is recovered by elastic recoil of the particles. Modeling viscoelastic recoil by a Voigt body, in which a viscosity *η *and the rigidity modulus *ν *are in parallel (Appendix), a direct estimation of the elasticity modulus is possible. In a Voigt-body, elastic recoil returns the dipoles back to their orientation before stress application. Particle twisting in living macrophages does not yield complete recoil (Figure 5); most of the strain remains non-recoverable. This required an extension of the Voigt-body by an additional viscous element *η*_1_. The whole system is then called a Voigt-Maxwell body. Non-recoverable strain can indicate non-reversible deformations in the cytoplasm.

Cell stiffness was estimated as the ratio between mean stress to strain after a constant twisting duration of 10 sec.:



This analysis of stiffness does not view for specific viscous or elastic properties. Therefore this parameter provides an integral description of the cytoskeletal mechanical properties.

### Data analysis

Because of the influence of phagocytosis and macrophages activation on the estimation of macrophage motility and mechanical cytoskeletal properties only the data from one week after particle deposition till the end of the study were used to form mean values for cell motility and cytoskeletal mechanics. This also eliminates possible effects of particles being deposited in the airways. The SAS statistical software was used for data analysis. Because some parameters were not normally distributed, data reported in Table 1 and Table 2 were compared using a t-Test and a non-parametric Wilcoxon-test. Significance levels obtained with the Wilcoxon-test were mostly higher compared to the t-Test. The border of achieved significance given in the results holds for both tests, when not separately mentioned. Pearson's and Spearman's rank correlation analyses were performed in order to test possible relations between the parameters.

**Table 2:** Results of the measurement of relaxation and of randomization energy, E_r_

	Healthy	SAR	FIB	COB
N	17	15	12	19
A_r_s_	0.76 +/- 0.04	0.79 +/- 0.03 (**)	0.79 +/- 0.07 (ns)	0.75 +/- 0.06 (ns)
T_r_f_, s	20.6 +/- 7.0	14.5 +/- 3.8 (**)	14.2 +/- 5.6 (**)	16.9 +/- 5.4 (ns)
T_r_s_	259 +/- 61	257 +/- 39 (ns)	221 +/- 47 (ns)	239 +/- 57 (ns)
T_r_a_, s	66.1 +/- 16.3	57.5 +/- 9.1 (ns)	50.2 +/- 12.5 (**)	55.7 +/- 14.5 (*)
b1	0.61 +/- 0.05	0.63 +/- 0.02 (ns)	0.60 +/- 0.04 (ns)	0.59 +/- 0.07 (ns)
b5	0.23 +/- 0.07	0.24 +/- 0.04 (ns)	0.20 +/- 0.06 (ns)	0.21 +/- 0.07 (ns)
E_r_, 10^-18 ^J	6.0 +/- 2.1	4.6 +/- 1.5 (*)	5.2 +/- 2.6 (ns)	5.4 +/- 3.1 (ns)

Data are presented as mean +/- SD; SAR: patients with sarcoidosis, IPF: patients with idiopathic pulmonary fibrosis, COB: patients with chronic obstructive bronchitis, N: number of subjects, parameters of the two-term exponential relaxation model: A_r_s_: fraction of slow decay, T_r_f_: time constant of fast decay, T_r_s_: time constant of slow decay, T_r_a_: time constant of initial decay; b1: normalized decay after 1 min.; b5: normalized decay after 5 min.; E_r_: cell energy; ns: not significant; *: p < 0.05, **: p < 0.01).

## Results

### Lung function data

Lung function data of the study groups are given in Table 1. Healthy smokers had normal lung function data (FEV_1 _= 108 +/- 13 % predicted). The study groups did not differ significantly from healthy subjects in their airway resistance (R_aw_). The severity of airflow limitation based on values of FEV_1 _(% predicted) was mild or moderate in the group of patients with COB, SAR and IPF. Patients with chronic obstructive bronchitis (COB) had a significantly lower FEV_1_/VC ratio and MEF_75-25 _than that of healthy subjects. In patients with SAR and IPF, vital capacity was significantly lower than that of healthy subjects. As for T_L,CO_, only patients with IPF differed significantly from healthy subjects.

### Macrophage motility (relaxation) in healthy and diseased subjects

Table 2 and Figure 2 show the results of relaxation in all investigated groups. In healthy subjects and in patients none of the relaxation parameters was significantly influenced by cigarette smoking. Therefore, in every group, the data of NS and S were matched together, allowing the direct relation of all relaxation data of the patient groups to the healthy subjects. Figure 2 gives a plot of the mean relaxation curve in every group. This curve is a function of two exponential terms (Eq. A2) and was calculated by the mean relaxation time constants of every group. The solid line describes the behavior in healthy subjects (Healthy) together with the relative decay after 1 min. and after 5 min., b1 and b5, and the corresponding standard deviation (SD). The mean relaxation behavior in patients (COB, SAR and IPF) is in the range of relaxation in healthy subjects. We can estimate in tendency a faster relaxation in patients with COB and with IPF.

More structural information can be obtained from the analysis of the two exponential- term relaxation model. In SAR, the fraction of slow relaxation (A_r_s_) is significantly increased (p = 0.01). In SAR and in IPF, there is an initially faster decay (smaller T_r_f_) compared to healthy (p < 0.01). In IPF, the slow relaxation is faster (smaller T_r_s_) in tendency (p = 0.07).

### Randomization energy E_r_

Cellular randomization energy estimated by the Langevin model is E_r _≈ 10^-18 ^J. This value is much higher than thermal energy (kT = 4.2·10^-21 ^J at 37°C) and shows that thermal energy can be neglected in the analysis of intracellular phagosome motions. In vitro investigations have shown that E_r _is correlated with the energy (ATP) status of the macrophages [39]. The hydroxylation of one ATP molecule yields an energy of ≈ 10^-19 ^J. Cellular energy E_r _represents the motor of relaxation and phagosome transport in the hydrodynamic relaxation model. In healthy subjects, the cell energy E_r _is not influenced by cigarette smoking, allowing the matching of NS, S and XS data. The data of all investigated groups are shown in Table 2. Compared to healthy subjects, E_r _is lower in SAR (p < 0.05). In IPF and COB, E_r _is lower in tendency.

### Continuous particle twisting

**Figure 6:**
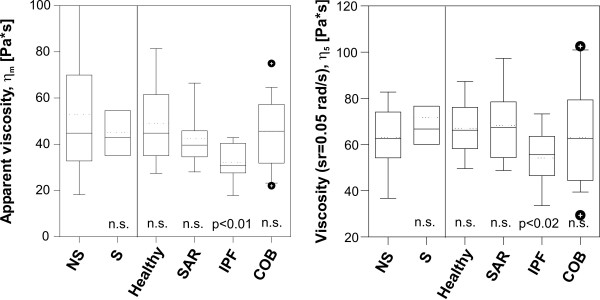
Box plot of the initial apparent viscosity (*η*_m_, a) and the apparent viscosity at a shear rate of 0.05 rad/s (*η*_5_, b) after continuous particle twisting for non-smoking (NS) and smoking (S) healthy subjects and for patients with the investigated lung diseases (dashed lines denote median values).

The results of the continuous particle twisting in alveolar macrophages are summarized in Figure 6. Apparent viscosity *η*_m _(after 5 sec particle twisting) is not different between NS and S in healthy subjects, as well as in the patients groups (Figure 6a). Therefore in every group, the data of NS, S, and XS were matched together. In patients with SAR and COB, apparent viscosity shows no differences to healthy subjects. Apparent viscosity *η*_m _is significantly lower in patients with IPF (both in IPF(NS) and in IPF(S), p < 0.01). The cytoskeleton of IPF macrophages is softer compared to healthy macrophages.

The increase of apparent viscosity *η *with decreasing shear rate sr according to the power law *η *~ sr^-*α *^(Figure 4) could be verified in all measurements. The power was *α *= 0.98 ± 0.02, implying a linear increase of apparent viscosity with decreasing shear rate. The power *α *was not influenced by cigarette smoking or by disease. Using the power law behavior, the normalized viscosity *η*_5 _at a shear rate of 0.05 rad/s was estimated for all subjects and is shown in Figure 6b. The principal behavior of *η*_5 _in healthy subjects and in patients is not different to *η*_m _(Figure 6a), but the data have a smaller variation.

### Discrete particle twisting

**Figure 7:**
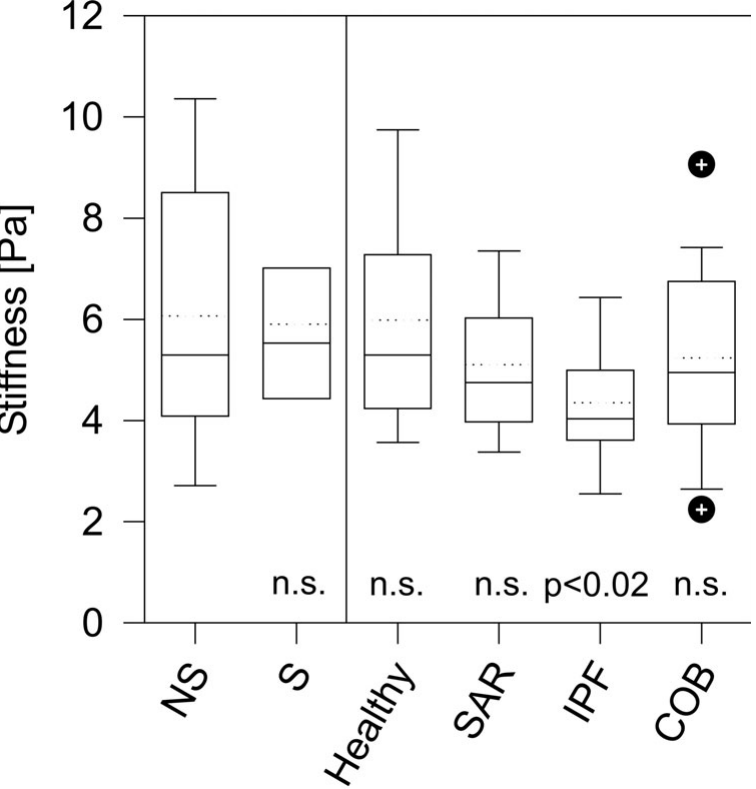
Box plot of cytoskeletal stiffness (ratio between mean stress and strain) after 10 s discrete particle twisting for non-smoking (NS) and smoking (S) healthy subjects and for patients with lung diseases (dashed lines denote median values).

More structural information on the mechanical behavior of the cytoskeleton is obtained from discrete particle twisting when the twisting field was applied for 10 sec. In healthy subjects, cigarette smoke consumption does not influence any of the parameters of discrete particle twisting, allowing the matching of the NS, XS and S data in each group. In healthy subjects, mean cytoskeletal stiffness was 6.0 ± 2.2 Pa after 10 sec. particle twisting (Figure 7). 72 ± 13 % of the rotational strain was not recoverable, which may result from pure viscous shear (or permanent deformation). Elastic recoil was only visible in the remaining 28% of particle shear and had a time constant of T_erc _= 30 ± 14 sec. The corresponding units of Voigt-Maxwell model according to Figure 5 and Equations A7 and A8 reveal a mean viscosity of *η*_1 _= 97 ± 58 Pa·s for the pure viscous compartment and a viscosity of *η*_2 _= 166 ± 53 Pa·s and an elasticity modulus of *ν*_2 _= 6.3 ± 2.7 Pa for the viscoelastic compartment.

Cell stiffness is not different in patients with COB compared to healthy, as shown in Figure 7. The pure viscous shear reveals results comparable to healthy subjects, but the viscoelastic compartment shows a faster elastic recoil (T_erc _= 17 ± 6 s, p < 0.01), which may originate from a significantly lowered viscosity in this compartment (*η*_2 _= 107 ± 42 Pa·s, p < 0.01). Finally, the elasticity modulus *ν *remains unchanged compared to healthy.

Patients with SAR show no modifications in the integrating parameter cell stiffness. The shear strain in the pure viscous compartments yields a viscosity comparable to healthy subjects. The viscoelastic compartments has a faster elastic recoil (T_erc _= 20 ± 7 s, p = 0.01), which is not correlated with a (compared to healthy subjects) modified viscosity in this compartment, but the elasticity modulus is increased in tendency.

In patients with IPF, cytoskeletal stiffness is significantly reduced (4.2 ± 1.2 Pa, p < 0.02), as shown in Figure 7. This reduced stiffness primarily results from a decreased viscosity of the pure viscous compartment (*η*_1 _= 61 ± 15 Pa·s, p = 0.02). The viscoelastic compartment (including elastic recoil time constant) is not altered in patients with IPF compared to healthy. This is in agreement with continuous particle twisting where apparent viscosity was lowered.

## Discussion

### Effect of cigarette smoking in healthy subjects

Relaxation shows no differences between healthy NS and S, although there seems to be in tendency a slower decay with a reduced motility in healthy smokers. This result is surprising because we expected a strong influence of cigarette smoking on AM motility and on relaxation. Two in vitro studies on relaxation in smokers and non-smokers have been done [22-24]. In both studies, alveolar macrophages were harvested by bronchoalveolar lavage (BAL) from the human lungs and then incubated together with magnetic micro particles for 24 hours. Relaxation was measured in a cytomagnetometric system. In Valberg's study, relaxation was faster in smokers compared to non-smokers. In this study, the cigarette smoke consumption was not clearly reported, but the subjects seem to be younger (20 – 30 years of age). In the second study by Gehr and Im Hof, relaxation did not show differences between NS and S. The subjects in this study had a mean cumulative cigarette smoke consumption of 8 PY, suggesting that they are younger. In agreement to Gehr's and Imhof's study our data suggest that even with a high cigarette smoke consumption of 45 PY cell organelle motions are not affected. We have to keep in mind that our data do not report the response of an acute cigarette smoking. In smokers there seems to be an adaptation and the primary response of the lung to the permanent cigarette smoke exposure is the increase in the number of macrophages in the lungs. The differences found in one of the *in vitro *studies are probably influenced by the BAL procedure and by the incubation method.

In agreement to the relaxation behavior in relation to cigarette smoking is the result for the driving energy of relaxation. Cellular energy E_r _was not influenced by cigarette smoking. The significantly lower E_r _in SAR and in tendency in IPF and in COB may account for effects on the energy status and metabolism of macrophages.

Additionally, cigarette smoking did not influence any of the mechanical properties estimated by continuous or by discrete particle twisting. The results suggest that cigarette smoking does not influence intracellular phagosome transport processes, (relaxation), which reflects macrophage motility, nor directed phagosome motion (magnetic twisting), which reflects the cytoskeletal integrity. The biochemical and immunological response of the lungs to chronic cigarette smoking has no correlation to the cell motility and mechanical integrity of the cytoskeleton of macrophages.

### Phagosome motion and twisting in lung diseases

Although it is known from biochemical and immunological investigations that macrophages are activated in SAR, and specifically during exacerbations, such as enhanced proliferation activity [40]; enhanced release of tumor necrosis factor alpha (TNF *α*) and interleukin-1 (IL-1) [41], the course of relaxation (b5) does not differ significantly from healthy subjects, only the initial decay was accelerated. In addition E_r_, the driving energy of relaxation, was lower in SAR, which is opposite to expectations from a faster initial relaxation, suggesting an increase in E_r_. But the faster initial relaxation in SAR correlates with the faster elastic recoil after discrete particle twisting, which may reveal altered elastic properties of the cytoskeleton. The data show that the rheological properties of the cytoskeleton have significant impact on phagosome motion and on relaxation. We have previously shown that cytoskeletal elasticity is primarily related to the actin cytoskeleton [16], suggesting that the SAR may influence the microfilamentous structure of the macrophages. The limited effects of the disease SAR on the cytoskeletal dynamics may result in part from the stable status of the disease during the investigation period and the use of specific drugs.

In COB, a faster initial relaxation was recorded, which correlates with the faster elastic recoil in discrete particle twisting. The compartmental analysis showed that reason for this faster recoil is a lowered viscosity in the viscoelastic compartment (*η*_2_).

The faster relaxation in IPF correlates with significant alterations in the mechanical integrity of the cytoskeleton of the macrophages. This is verified both in continuous and in discrete phagosome twisting, where all viscosities and the stiffness of the cytoskeleton are lowered. We conclude that IPF coincides with a structural damage of the cytoskeletal integrity, resulting in multiple cytoskeletal dysfunctions. *In vitro*, such damage can be induced by cytoskeletal drugs, such as Cytochalasin D, which disrupts microfilaments, Nocodazole, which disrupts microtubuli, or Acrylamide, which disrupts intermediate filaments [16,42]. Destruction of cytoskeletal filaments of macrophages can cause several dysfunctions, such as retarded migration and phagocytosis, together with a retarded cytotoxicity against bacteria or viruses, besides other immunological and biochemical dysfunctions. Comparable inhibition of cytoskeletal functions was shown after incubation of macrophages with toxic particles [43], or with different types of ultrafine environmental particles, resulting in a impairment of relaxation, and a stiffening of the cytoskeleton [20], and it could be shown that the calcium metabolism plays an important role in this dysfunctions [21]. The significant damage in cytoskeletal structure and function may explain the impaired phagocytosis [17] and impaired alveolar clearance [11] in IPF patients.

### Analysis of correlation

Estimation of the relaxation parameters, cellular energy, cumulative, and discrete particle twisting are separate measurements. A correlation analysis of all data provides information about the interconnection of parameters and can be used to test the proposed rheological models. The Voigt-Maxwell system used in this study is the simplest configuration giving characteristic features.

#### Macrophage motility

The randomization energy E_r _is the motor of relaxation. Therefore, a strong correlation between the relaxation time constants and E_r _was expected, which can not be verified by the data. E_r _shows a strong correlation with the viscosity parameters, both estimated from discrete as well as from continuous particle twisting, where the pure viscous compartment *η*_1 _is the relevant parameter (coefficient of correlation, cc = 0.64, p < 0.001). This suggests that the viscosity estimations are influenced by the intracellular phagosome transport mechanisms.

#### Continuous particle twisting

**Figure 8:**
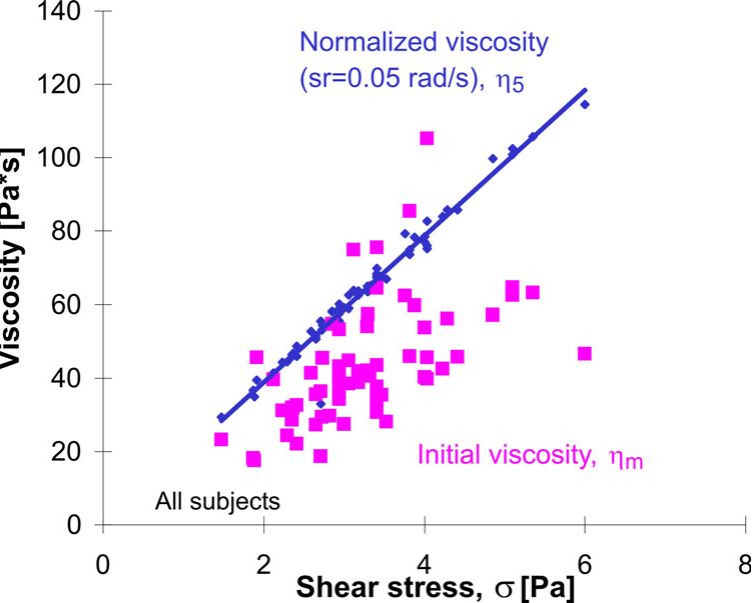
Correlation between viscosity and applied shear stress in continuous particle twisting.

It has been shown that the mechanical properties of the cytoskeleton are non-Newtonian; and the estimated viscosity depends on the shear rate and on the twisting force [15]. The cytoskeleton shows increased stiffening with decreasing shear rate. From the power law behavior, the viscosity at the shear rate sr = 0.05 rad/s, *η*_5 _was estimated for all subjects, thus removing the shear rate influence. This normalized viscosity *η*_5 _has a reduced variability compared to the initial viscosity *η*_m_, but still reports the dysfunctions in IPF. *η*_5 _is highly dependent on the mean stress applied during particle twisting, as shown in Figure 8 (cc = 0.985, p < 0.001). The behavior describes a stiffening of the cytoskeleton, not only with decreasing shear rate, but also with increasing stress, which is not the case for *η*_m _(Figure 8). Such stiffening was found previously in mechanical studies of endothelial cells [42] and also in alveolar macrophages [15]. This linear stiffening behavior is typical for living tissues [44] and is evidence for an intact cytoskeletal network. Such a stiffening cannot be described by a rheological model and implies a cytoskeleton structure of interconnected units, which was proposed by the tensegrity model [45,46].

#### Discrete particle twisting

**Figure 9:**
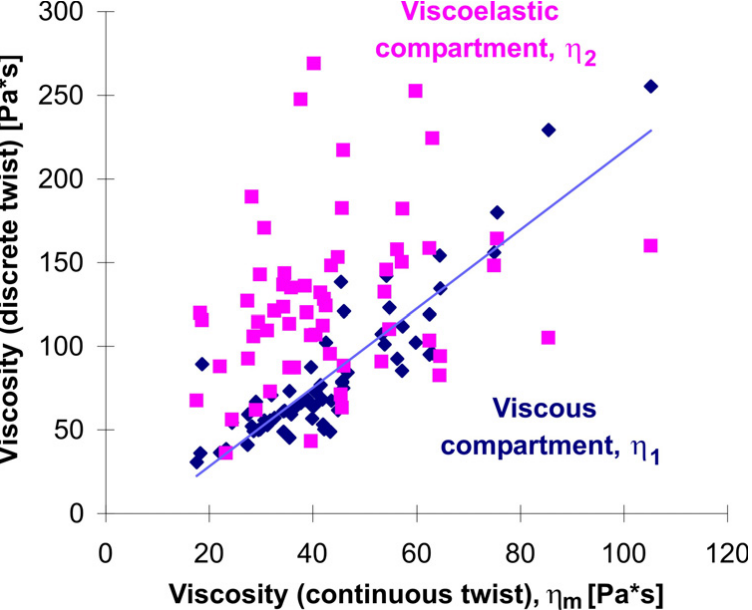
Correlation between viscosity estimations after discrete and after continuous particle twisting.

As mentioned above, discrete particle twisting reflects two different rheological compartments. Particle twisting in the pure viscous unit bears no energy storage and shows no strain recovery. The second compartment is a viscoelastic body, in which all strain is recoverable. Figure 9 shows the correlation between both viscosity estimations from discrete particle twisting with the apparent viscosity from continuous particle twisting. The pure viscous compartment *η*_1 _correlates with the apparent viscosity *η*_m _(cc = 0.87, p < 0.001), while the viscosity of the viscoelastic compartment *η*_2 _does not. The pure viscous compartment *η*_1 _is determined by the main fraction of shear (72 +/- 13%); the viscoelastic compartment is of secondary influence. Variations in cytoskeletal stiffness are primarily mediated by the pure viscous compartment.

Since the viscous shear is dominant in discrete particle twisting and the slow decay is the main relaxation behavior, we suggest that both phenomena result from the same hydrodynamic and cytoskeletal unit, the viscosity *η*_1_. But in the correlation analysis, the two parameters T_r_s _and *η*_1 _appear to be independent. None of the relaxation parameters correlate with any of the twisting parameters. These results suggest that relaxation (cellular phagosome motion) and magnetic particle twisting (directed external phagosome motion) may reflect two independent mechanisms within the cell dynamics. Relaxation measures the transport of phagosomes along cytoskeletal units, while magnetic particle twisting measures the mechanical integrity (viscoelasticity) of the cytoskeletal units via the application of external forces.

There are several indications that vesicles and phagosomes are primarily coupled to the microtubules [47]. Our data are in agreement with these studies and suggest that the pure viscous compartment of discrete particle twisting reflects the mechanical behavior of microtubules. Because microfilaments appear highly viscoelastic [48,49], the viscoelastic compartment may be associated with the microfilaments.

Although the role of the different cytoskeletal substructures during a defense reaction in the lung is not understood, we can conclude that the method delivers information about basic physiological processes in macrophages. Microfilaments mediate phagocytosis and the formation of phagosomes, and the microtubuli provide the structure for phagosome trafficking and phagosome – lysosome fusion. Therefore MPG investigations deliver *in vivo *information about the function of macrophages and might help to interpret pathophysiological reactions in macrophages in the lungs.

Another feature of MPG might be an *in vivo *monitor of the action of toxic pollutants and of drugs on macrophages in the lungs.

## Appendix

### Hydrodynamic model of relaxation

A first step to model relaxation is to describe the stochastic particle twisting as rotational Brownian motion. In this model, thermal energy kT induces random rotational pulses on the particles that cause a stochastic twisting of the magnetic dipole particles. These rotational movements happen within a viscous environment (viscosity *η*). We apply this model to the intracellular rotation of particle containing phagosomes in macrophages, where kT is replaced by some unknown cellular energy E_r_. When using spherical particles, the decay of the lung field (LF) follows an exponential function according to [36]:



where V is the volume of the particles, *η *is the viscosity of the cytoplasm, and *κ *is the rotational shape factor (*κ *= 6 for spheres). An exponential relaxation could be verified using monodisperse spherical magnetic particles, which were suspended in a Newtonian viscosity [38].

*In vivo *and *in vitro *investigations of relaxation with spherical particles in macrophages yield deviations from a single exponential decay. An initially fast decaying phase (over the first 30 s) was followed by a slower exponential phase lasting up to 20 min. Therefore, the decay was fitted by a double exponential function:



where T_r_f _and T_r_s _are the time constants for the fast and the slow relaxation phases, respectively; A_r_s _describes the fraction of relaxation following the slow phase. About 20% of the LF followed the fast decay with a half-time of 20 sec., while 80% of the LF followed the slowly decay with half-times near 200 sec. Further studies have shown that both the fast and the slow decaying fraction depend on the particle size [38].

### Particle twisting in a magnetic field (secondary magnetization)

Secondary magnetization describes the twist of the dipole particles in a weak magnetic field B_M_. A magnetic torque N_mag _= m B_M _sin*θ *acts on a remanent dipole m in an external magnetic field B_M _that twists the particle until the dipole is aligned with the magnetizing field: *θ *is the angle between B_M _and m. The viscosity *η *of the surrounding fluid resists this twist, resulting a hydrodynamic retarding torque N_hyd _= -*κ*V*η*·d*θ*/dt; d*θ*/dt is the angular velocity of rotation (shear rate), V is the volume and *κ *is the shape factor of the particle (*κ *= 6 for spheres). The balance of both torques describes the dipole particle rotation:



with the solution:



M_T _= m/V is the remanent magnetization of the particles, *θ*_0 _is the initial orientation of the dipole m. Only the component of magnetic moment parallel to the magnetizing field, m·cos*θ*(*θ*_0_,t), is detected. From Eq. A4, cos*θ*(*θ*_0_,t) can be expressed as:



The time constant *τ *(Eq. A4) shows that particle twisting is independent of particle size. This method of viscosity measurement was calibrated with magnetite spheres suspended in a highly viscous Newtonian fluid [38].

Equations A3 – A5 only hold for Newtonian viscosities, where the viscosity is independent of the shear rate. Including elastic properties makes the system non-Newtonian. Nevertheless, secondary magnetization measurements can be analyzed with Eq. A3, yielding a shear rate dependent apparent viscosity. Since the relative LF is a measure of cos*θ*(t), the orientation angle *θ*(t) = arccos(B(t)/B_max_) and the shear rate Δ*θ*/Δt can be predicted. Then Eq. A3 is used to estimate the apparent viscosity as:



### Viscoelastic recoil

The relaxation and particle twisting measurements have shown that the viscosity of the cytoskeleton behaves non-Newtonian and has elastic properties. The simplest viscoelastic body is a *Voigt-body*, where the viscosity *η *is in parallel with an elastic element with rigidity *ν*. The differential equation for this simple viscoelastic system is:



*θ*_0 _is the orientation before particle twist. During particle rotation in a viscoelastic Voigt-body, elastic energy is stored. After removing the magnetic torque (B_M _= 0), elastic recoil happens. Eq. A7 predicts elastic recoil behavior (*σ *= 0) as:



T_erc _is the time constant of elastic recoil. Knowing the viscosity *η*, the rigidity modulus *ν *can be estimated from the time path of the elastic recoil process. In a Voigt-body, elastic recoil returns the dipoles to their orientation before stress application, independent of applied stress.

The experimental results show this behavior only for weak twisting fields [15]. Particle twisting with higher stress does not yield complete recoil; some non-recoverable strain remains. Non-recoverable strain might indicate permanent deformations in the cytoplasm. For a rheological description, the Voigt-body was extended by an additional viscous element. The whole system is called a Voigt-Maxwell body.

## Authors' contributions

WM, WB and WGK performed the biomagnetic investigations, such as generation of particles and inhalation, the biomagnetic measurements, and the data analysis. MK and KH performed the clinical part of the study, such as evaluation and characterization of the participants.
